# Coordinating attention requires coordinated senses

**DOI:** 10.3758/s13423-020-01766-z

**Published:** 2020-07-14

**Authors:** Lucas Battich, Merle Fairhurst, Ophelia Deroy

**Affiliations:** 1 Faculty of Philosophy and Philosophy of Science, Ludwig Maximilian University Munich, Geschwister-Scholl-Platz 1, Munich, 80359 Germany; 2 Graduate School of Systemic Neurosciences, Ludwig Maximilian University Munich, Munich, Germany; 3 Munich Center for Neuroscience, Ludwig Maximilian University Munich, Munich, Germany; 4 Institut für Psychologie, Fakultät für Humanwissenschaften, Universität der Bundeswehr München, Munich, Germany; 5 Institute of Philosophy, School of Advanced Study, University of London, London, UK

**Keywords:** Joint attention, Social cognition, Cross-modal attention, Multisensory perception

## Abstract

From playing basketball to ordering at a food counter, we frequently and effortlessly coordinate our attention with others towards a common focus: we look at the ball, or point at a piece of cake. This non-verbal coordination of attention plays a fundamental role in our social lives: it ensures that we refer to the same object, develop a shared language, understand each other’s mental states, and coordinate our actions. Models of joint attention generally attribute this accomplishment to gaze coordination. But are visual attentional mechanisms sufficient to achieve joint attention, in all cases? Besides cases where visual information is missing, we show how combining it with other senses can be helpful, and even necessary to certain uses of joint attention. We explain the two ways in which non-visual cues contribute to joint attention: either as enhancers, when they complement gaze and pointing gestures in order to coordinate joint attention on visible objects, or as modality pointers, when joint attention needs to be shifted away from the whole object to one of its properties, say weight or texture. This multisensory approach to joint attention has important implications for social robotics, clinical diagnostics, pedagogy and theoretical debates on the construction of a shared world.

## There is more to joint attention than meets the eye

Infant and caregiver coordinate their attention on a toy while learning its name; jazz musicians jointly attend to the music they play together, and hunters can jointly track the smell or sounds of prey in the forest. The ability to coordinate our perception on a shared object of interest comes to most of us between the ages of 9 and 18 months. In our everyday life, we continue to rely on this non-verbal skill, otherwise known as joint attention, to communicate, share experiences, and coordinate with others.

Joint attention has been proposed as one of the essential ingredients of social skills in humans (Adamson, Bakeman, Suma, & Robins, 2019; Carpenter, Nagell, Tomasello, Butterworth, & Moore, 1998; Eilan, Hoerl, McCormack, & Roessler, 2005; Moore & Dunham, 1995; Seemann, 2011; Tomasello & Farrar, 1986) and, arguably, across other animal species (Ben Mocha, Mundry, & Pika, 2019; Leavens & Racine, 2009). In most of these accounts, joint attention is measured through the capacity to follow gaze and pointing gestures and coordinate on visible targets (Mundy & Newell, 2007). But does coordinating on visible objects only depend on vision? And what happens when we need to coordinate, not on visible targets, but on auditory, tactile, or multisensory ones?

Uncontroversially, shouting or touching someone’s shoulder can be useful to make someone pay attention or orient in the right direction. The role of auditory or tactile alerting signals as accessory cues is well established in primate (Liebal, Waller, Burrows, & Slocombe, 2014) and non-primate (Ben Mocha et al., 2019; Bro-Jørgensen, 2010; Rowe, 1999) animal multimodal communication. It is similarly uncontroversial that non-visual senses often act as a *background* or mere enabling condition for visual attention (for instance, by using vestibular and proprioceptive cues to determine the spatial orientation of one’s body in the world, and orient visual attention accordingly). Existing work in the domain of joint attention would certainly accept that other sensory modalities are involved or that joint attention occurs in multisensory settings. Highlighting that joint attention is fundamentally a multisensory phenomenon, however, stresses that non-visual senses are not merely accessories to what could otherwise be defined as a visual phenomenon. Our goal is to provide a more systematic representation of how non-visual sensory resources contribute to joint attention. More specifically, we argue that non-visual senses play two crucial roles. First, they interact closely with gaze and pointing gestures to prime or *enhance* the coordination of visual attention. Non-visual senses can certainly act as distractors, having a negative impact on joint attention. In most cases, however, and with the exception of rare clinical or artificial cases, which we discuss below, other senses are at least minimally involved in the success of joint attention. Second, they play a *necessary* role when it comes to extending social coordination to non-visual and amodal properties of objects and events in the world.

Consider what would happen if gaze and pointing were indeed all there was to the coordination of attention: without computing information from multiple senses, either serially or in conjunction, our referential intentions would run a much higher risk of remaining ambiguous (see *Non-visual senses enhance visual joint attention*). We could not coordinate on non-visible and more abstract aspects of the world (see *Non-visual senses are necessary to extend joint attention*). The current multisensory account is better than a strictly visual one when it comes to explaining how joint attention establishes a socially shared world, where mind-independent objects can be attended in common (see *Theoretical implications: Sharing more than a visual world*). It also has implications for clinical settings and social robotics which are currently focused on gaze-following: with our new account, deficits in gaze coordination could potentially be compensated for by non-visual modalities, and social robots could coordinate attention with humans even without fine-grained gaze-following capacities (see *Applications: Multisensory strategies for the clinic, the school and social robotics*).

## Visual joint attention

When Jerome Bruner and colleagues introduced the term *joint attention* to the research on the ontogeny of communication (Bruner, 1974; Scaife & Bruner, 1975), they referred to infants’ developing capacity to share their experiences about objects and events with others, and learn word meanings. Now, the construct is used to explain many aspects of our social activities: joint attention in infancy predicts future social competence (Mundy & Sigman, 2015) and emotion regulation, and may reinforce executive functions (Morales, Mundy, Crowson, Neal, & Delgado, 2005; Swingler, Perry, & Calkins, 2015). For adults, engaging in joint attention modulates multiple cognitive abilities (Shteynberg, 2015), including working memory (Gregory & Jackson, 2017; Kim & Mundy, 2012), mental spatial rotation (Böckler, Knoblich, & Sebanz, 2011), and affective appraisals to objects in the environment (Bayliss, Paul, Cannon, & Tipper, 2006).

Bruner’s pioneering work centered on joint *visual* attention (Scaife & Bruner, 1975). By and large, subsequent research has remained exclusively focused on the visual domain. Gaze behavior can be easily measured and controlled in laboratory conditions and is therefore a powerful means to study joint attention. In arguing for a multisensory approach, we do not aim to diminish the important role played by gaze cues. Decades of research on gaze following and gaze alternation have firmly established their importance in development and cognition (Flom, Lee, & Muir, 2017; Frischen, Bayliss, & Tipper, 2007; Schilbach, 2015; Shepherd, 2010), and have provided a solid basis for the study of joint attention.

**Fig. 1:**
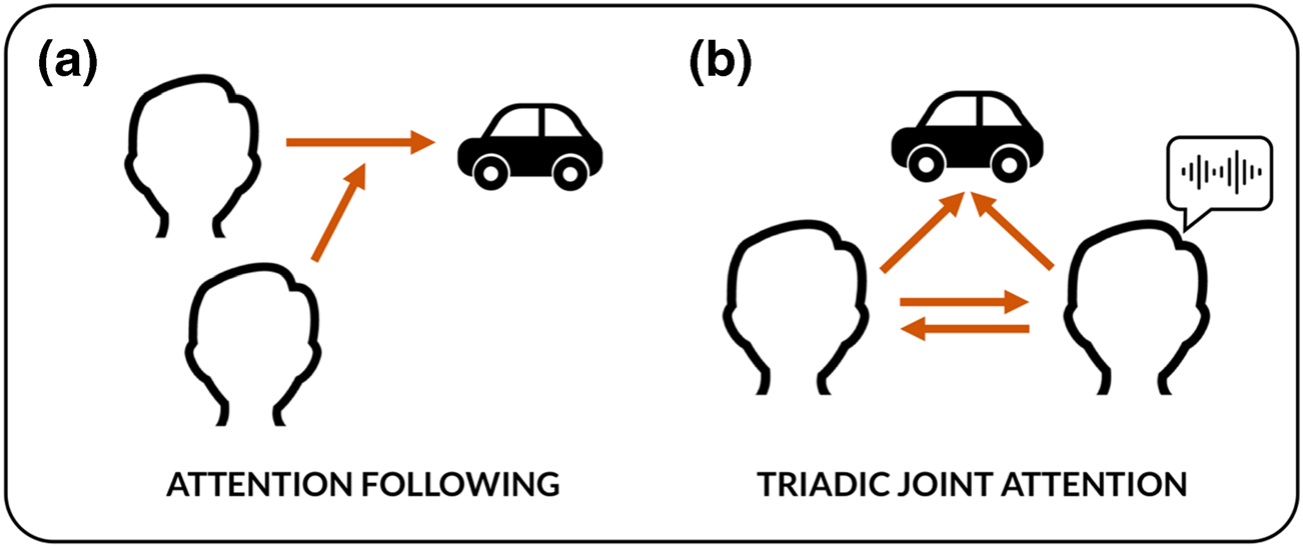
Following attention is different from coordinating attention. (**a**) Attention following is characterized by the unilateral response of one individual. It can consist of behaviors such as gaze following, or the monitoring of others’ bodily posture and gestures, and responding to vocal and haptic cues. Attention following is a pre-condition for full joint attention, and occurs earlier in development. (**b**) Coordination of attention is characterized by the reciprocal interaction between individuals toward a third object. In addition to gaze following, joint attention includes gaze-alternation and directing other’s gaze through pointing — but also other senses

Research into the early development of joint attention distinguishes between *responding* to joint attention by following the direction of others’ attention, and *initiating* joint attention by directing or leading the attention of others to a third object or event (Mundy & Newell, 2007). Responding to joint attention, sometimes considered equivalent to following someone’s perceptual cues, is the most studied form of joint attention (Fig. 1a) (Mundy, 2018; but see, e.g., Bayliss et al., 2013; Stephenson, Edwards, Howard, & Bayliss 2018). Whether following social cues for attention differs from following non-social cues like arrows remains a topic of debate and investigation, but uncontroversially engages spatial skills and perceptual gaze processing (Gregory, Hermens, Facey, & Hodgson, 2016; Hermens, 2017; Langton, Watt, & Bruce, 2000; Mundy, 2018; Shepherd, 2010). Senses other than vision can play an instrumental role alongside gaze and pointing gestures to guide spatial attention to visible objects.

Attention following, however, is often not sufficient for joint attention. For example, I can follow your attention without you noticing in any way that I did so, which would not count as joint attention. In addition to gaze following, joint attention requires the ability to engage in a *reciprocal coordination* that guarantees we are looking at the same object together (Mundy, 2018; Siposova & Carpenter, 2019) (Fig. 1b). This triadic coordination exhibits the understanding, even minimally, that both agents are mutually aiming at or aware of the object (Bakeman & Adamson, 1984; Mundy, 2016; Tomasello, 1995). Non-visual senses here may do more than facilitate attention following: they help to strategically select the appropriate target of joint attention between two individuals.

Engaging in joint attention requires one to know what one is attending to, as well as what the other is attending to. This in turn requires the combined processing of three types of information: (1) information about one’s own attentional state, including interoceptive and proprioceptive information (Mundy & Jarrold, 2010); (2) information about the other’s attentional state; (3) information about the target of joint attention (Mundy, 2018; Siposova & Carpenter, 2019). All three types of information and their processing can engage multiple senses, besides vision. Information about my own attention to the object of common reference may include whether I am actively handling the object, or merely looking at it. Information about the other’s attentional state will vary depending on whether they have access to the same sensory information I have. The strategies used to establish joint attention will vary when we coordinate on a smell, a sound, the color of an object, or a whole, complex multisensory event.

## Non-visual senses enhance visual joint attention

### Visual cues provide multisensory expectations

When processing information about the other’s attentional state, we can further distinguish between the sense I rely on to monitor the other's attention (e.g., I *gaze* at your hand grasping), and the sense they use, which I monitor to gather information about their attention (e.g., I gaze at your *hand grasping*). This distinction already pleads for the incorporation of richer sensory measures in models of joint attention than mutual eye contact, gaze following or gaze alternation. Observing someone’s touching actions, as well as someone being touched, activates similar neural circuits normally involved in the execution of those actions, and the processing of actual touch (Buccino et al., 2001; Keysers et al., 2004), suggesting that tactile expectations regarding the jointly attended object can be gathered vicariously even by sight alone. Studies have here looked at the use of coupled information from eye and hand gestures. When reaching and manipulating objects, gaze and hand movements are systematically coordinated with respect to the target object, with gaze fixation leading the subsequent hand movement (Horstmann & Hoffmann, 2005; Pelz, Hayhoe, & Loeber, 2001). This eye-hand coupling can provide a path for well-coordinated rapid and successful joint attentional interaction: although gaze provides a faster cue to the spatial area where the target is located, the hand trajectory while reaching and grasping provides a slower but more spatially precise and stable cue to the target’s location (Yu & Smith, 2013). Additionally, in following a grasping gesture, observers are sensitive to both the direction and the grip aperture size of the reaching hand to facilitate target detection (Tschentscher & Fischer, 2008). Reliance on multiple senses and their interaction may here help provide richer spatial and temporal representations of our environment (Keetels & Vroomen, 2012; Stoep, Postma, & Nijboer, 2017). These multisensory strategies are present during infant–caregiver joint attentional engagement, which reflects the multisensory nature of parent–infant dyadic communication (Gogate, Bahrick, & Watson, 2000; Gogate, Bolzani, & Betancourt, 2006; Hyde, Flom, & Porter, 2016). Multimodal behaviors help sustain joint attention between parents and infants from 12 to 16 months old, in particular when parents express some interest in an object looking at, talking about, and touching the jointly attended object (Suarez-Rivera, Smith, & Yu, 2019). One-year-old infants do not tend to follow the partner’s gaze to monitor their attention while playing together with a toy. Instead, they follow their hands (Yu & Smith, 2013). Taken together, this evidence suggests that non-visual senses and multisensory expectations are exploited in joint attention, especially to narrow down the spatial location of the target of joint attention through spatial redundancy.

Recent research on the emergence of pointing gestures reinforces this suggestion. Children interpret pointing gestures as if they were attempts to touch things (O’Madagain, Kachel, & Strickland, 2019), indicating that understanding visual cues about someone's touch toward a third object are ontogenetically prior to the understanding referential pointing gestures. This recent work suggests new methods to explore whether a similar relation is present in the phylogeny of grasping and pointing cues.

### Non-visual cues enhance visual target detection

**Fig. 2:**
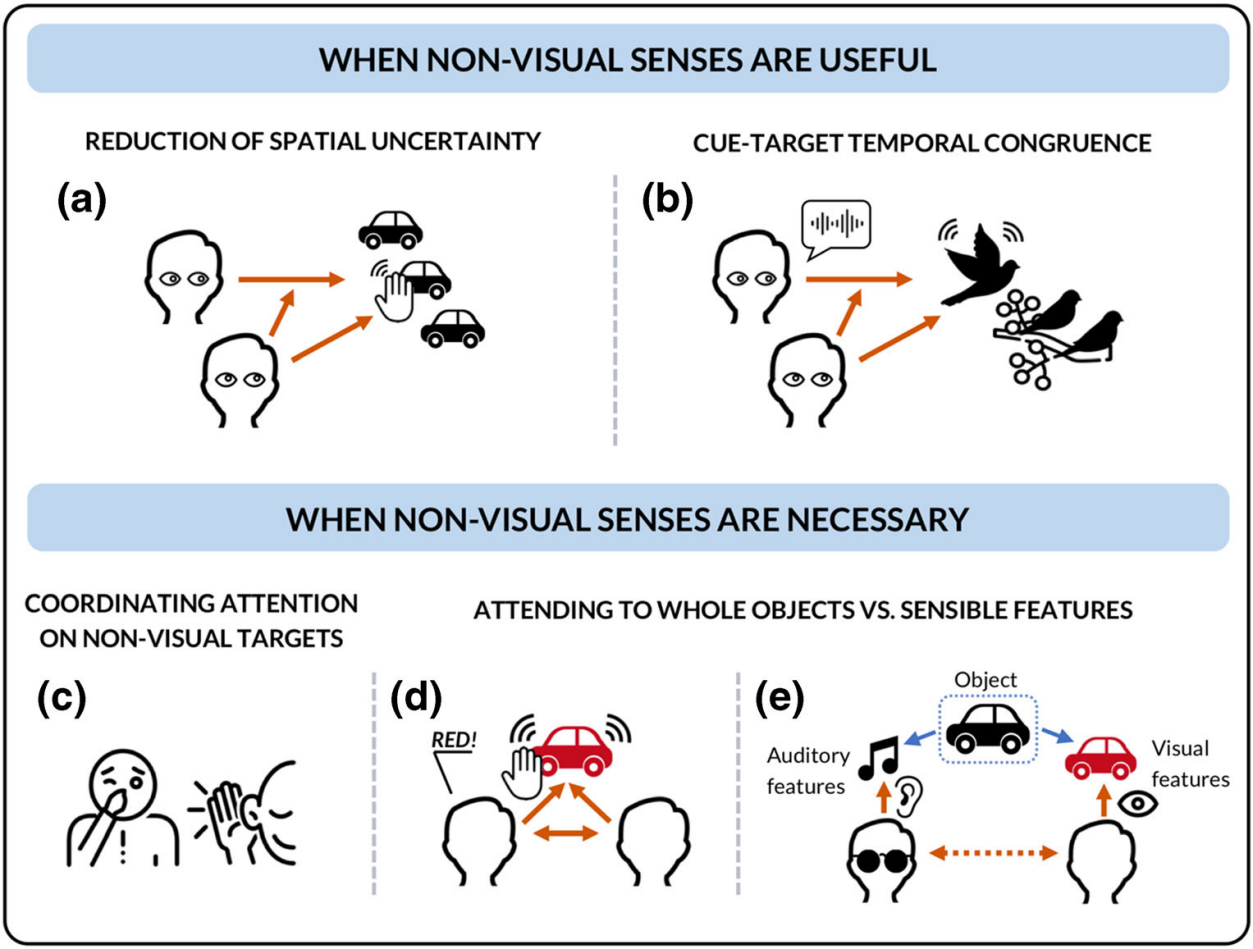
(**Upper panel**) Non-visual cues can complement visual cues in joint attention. (**a**) Redundant information delivered across modalities can increase accuracy and speed in following spatial cues: by monitoring someone’s eye-gaze cues in combination to their hand-grasping actions, the follower’s response in localizing the object of joint attention is enhanced. (**b**) Using temporal congruence between a cue and a target in different modalities to facilitate someone’s orienting to the correct visual target. (**Lower panel**) Non-visual cues are often necessary for joint attention. (**c**) Establishing joint attention toward a non-visual target by using ostensive visual cues: ostensive pointing at the relevant sensory organ (touching one’s ear or one’s nose) can provide evidence to another agent of the intention of attending to a non-visual stimulus (a sound, a smell). Such strategies rely on cognitive abilities to infer that the target is non-visual. (**d**) Exploiting temporal synchrony: a parent shakes an object in a temporally synchronous manner congruent with their uttering the word “red.” While the visual stimulus and the auditory stimulus have different causal sources (the toy and the parent), the information is conveyed that the word “red” is associated with a visual property of the toy. (**e**) Coordinating on objects we each experience through different modalities: each subject must process information about each other’s modal access relative to the target to successfully achieve coordination

Joint attention can be established through gaze alone (Flom et al., 2017). In many social contexts, the use of visual cues can be sufficient to coordinate attention, but may not always be the most *efficient*. In information theory, adding redundancy to the initial message so that several portions of the message carry the same information increases the chance that the message is accurately received at the end of a noisy channel (Shannon, 1948). This is also true in perception. For an everyday illustration, consider trying to hit a nail with a hammer. It is possible to push the pointy part of the nail in the wall and then hammer it while relying only on vision, but by holding the nail with one hand, you can gather information about the nail’s spatial position both through vision and through your hand position. Studies in multisensory perception demonstrate that redundant information delivered across several sensory modalities increases the reliability of a sensory estimate: it enhances a perceiver’s accuracy and response time to detect the presence of a stimulus and to discriminate and identify a sensory feature (e.g., an object’s shape or its spatial location), a so-called *redundant-signals effect* (Ernst & Banks, 2002; Miller, 1982). It is safe to assume that redundancy of information across modalities is also usefully exploited when establishing and sustaining joint attention. For example, the caregiver will point to a toy car that the infant can see, and tap on the toy to make a noise. Here, the combination of the visual and auditory information enhances the infant’s accuracy and speed in shifting spatial attention (cf. Partan & Marler, 1999) (see Fig. 2a). In this section we review how multisensory information facilitates visual coordination and target detection, focusing on three mechanisms: spatial congruency, temporal synchrony, and cross-modal correspondences.

*Redundancy of spatial information* is shown to help with the orienting of visual attention in experiments where individual perceivers are presented with a task-irrelevant cue on the same or opposite side of the subsequent visual target. Participants tend to respond more rapidly, and more correctly, to visual targets appearing at the same location as the former task-irrelevant cue, rather than on the opposite side. This works for visual irrelevant cues (Posner, 1980; see Carrasco, 2011; Wright & Ward, 2008, for overviews) and also occurs across modalities: participants are faster and more accurate at detecting target stimuli in one modality when a task-irrelevant cue is presented in the same or similar location (McDonald, Teder-Sälejärvi, & Hillyard, 2000; Spence, McDonald, & Driver 2004a; see Talsma, Senkowski, Soto-Faraco, & Woldorff, 2010, for a review). This evidence suggests that when participants direct their spatial attention to a certain location driven by one modality, their sensitivity to stimuli in that location is also enhanced for other modalities. While these traditional cross-modal attention studies use nonsocial stimuli, there is growing evidence of similar effects with social ones. Gaze-cueing experiments using covert orienting paradigms have shown that cues from another’s gaze behavior facilitate the processing of tactile stimuli at the body location corresponding to the other’s gaze direction (Soto-Faraco, Sinnett, Alsius, & Kingstone, 2005). Recent work shows that gaze-based cues enhance the processing of tactile (De Jong & Dijkerman, 2019) and auditory (Nuku & Bekkering, 2010) stimuli at what is meant to be the jointly attended location. The current evidence of cross-modal effects in spatial attention gives us reason to think that a wide array of sensory cues, besides someone’s gaze or gesture direction, can be exploited to assist spatial coordination between joint attenders.

*Temporal synchrony* between cross-modal cues, in the absence of spatial congruence, also directs someone’s spatial attention. Van der Burg et al. (2008, 2009, 2010) have shown that the presentation of a spatially irrelevant cue in the auditory or tactile modality can facilitate a participant’s visual search performance in an environment with color-changing elements, when the non-visual cue is presented at the same time as a color change in the target element. Known as the “pip-and-pop effect,” these studies show that even when one sensory cue does not carry relevant spatial information, it can enhance the salience of a spatially relevant cue in a different modality (Ngo & Spence 2010). These cross-modal effects could be exploited in trying to establish joint attention to a target in a changing, dynamic environment. Touching someone's shoulder or vocalizing in synchrony with a certain movement or event (e.g., every time a particular bird jumps from a branch or flutters its wings) may be a better strategy to coordinate attention to it than pointing alone (Fig. 2B).

Finally, *the properties of the non-visual social cues* can also shape congruency effects, besides providing spatial or temporal congruence with visual cues. We are not talking here of semantic congruence (saying “dog” or “woof” while pointing at the visible dog) but of sensory congruence between properties such as pitch or loudness, and visual properties, such as brightness, shape, etc. Humans, like some other animals (Bee, Perrill, & Owen, 2000), exploit the environmental regularities that exist between sensory cues across modalities for communicative purposes. Such regularities show up in cross-modal correspondences, i.e. robust associations between independent features or dimensions across modalities (Spence, 2011; Spence & Deroy, 2013). For example, high-pitched sounds correspond to high spatial positions of a visual stimulus, so that when both features are congruently matched, attentional orienting to a target visual cue is facilitated (Bernstein & Edelstein, 1971). Other cross-modal correspondences, such as the one that exists between pitch and brightness, work together with temporal synchrony to elicit a “pip-and-pop effect” during visual search: when a visual target changes brightness, a congruent change in pitch of a task-irrelevant auditory cue enhances correct target detection (Klapetek, Ngo, & Spence 2012). The effects of cross-modal correspondence have so far been mostly studied in nonsocial domains. We suggest that they are also relevant in social domains. For example, when trying to direct your attention to an animal hiding in the trees, emitting a high-pitched rather than a low-pitched interjection might help direct attention to the higher part of the scene. To test this suggestion, future work on multisensory joint attention will have to address the role of cross-modal alerting signals, and how the processing of cross-modal social signals compares to nonsocial situations.

Importantly, how much spatial, temporal, and cross-modal congruence facilitate the processing of visual gaze or pointing gestures is ripe for more precise measurements, notably by artificially manipulating the discrepancy between the cues, and measuring the subsequent effects on joint attention.

### The interplay between coordinated attention and multisensory processing

Multisensory cues can help the social coordination of attention. Surprisingly, the reverse can also be true. A few innovative studies give evidence that coordinating attention with a partner modulates a participant’s multisensory processing. People are better able to ignore task-irrelevant stimuli in a distracting modality when they know that someone else is attending to these distractors (Heed, Habets, Sebanz, & Knoblich, 2010; Wahn, Keshava, Sinnett, Kingstone, & König, 2017).

In the first study (Heed et al., 2010), participants had to judge whether a tactile stimulus was presented on the upper or lower part of a cube, while a distractor visual stimulus was presented synchronously at the same or opposite elevation. In the individual task, participants responded faster and more accurately when the distractor stimulus was presented at the same elevation as the tactile target, showing a performance difference known as the cross-modal congruency effect (CCE; see Spence, Pavani, Maravita, & Holmes, 2004b, for a review). Interestingly, the CCE was significantly reduced when a partner was instructed to attend to the visual stimuli, indicating that participants could better ignore incongruent distractors when their partner responded on them. This effect was recently replicated in an audiovisual congruency task (Wahn et al., 2017) involving visual flashes and auditory tones originating from the same or opposite spatial vertical location. Knowing that someone else was attending to the incongruent flashes allowed participants to respond faster to the tones, resulting in a reduced CCE.

These studies show that responding jointly reduces the interference of competing stimuli in a multisensory setting (Wahn & König, 2017). The results seem at odds with a recent tradition of research showing that acting jointly increases the interference of irrelevant stimuli, presumably due participants co-representing each other’s tasks besides their own (Sebanz, Knoblich, & Prinz, 2003, 2005). For example, performing an object-based visual attention task jointly impairs performance (Böckler, Knoblich, & Sebanz, 2012), and the increase in interference of irrelevant information is well documented in Go/No Go joint Simon tasks (Dolk et al., 2014). The difference between the reduction and the increase of irrelevant interference in different joint attentional tasks may be due to the nature of the tasks studied. An efficient division of labor can be allowed when the different target stimuli of each co-actor’s task are presented concurrently, whereas the beneficial effect of filtering irrelevant information disappears when the task involves two competing Go/No Go actions (Dolk & Liepelt, 2018; Sellaro, Treccani, & Cubelli, 2018).

So far, studies have focused on coordinated social attention to separate cross-modal targets. Each participant attends and responds to a different modal stimulus, which facilitates a perceptual division of labor. A multisensory approach to joint attention should encourage us to extend this work to situations where partners attend and respond to the same multisensory stimuli, or try and ignore distractors in the same modality while focusing on another one. For example, when two subjects jointly coordinate their attention toward sounds and flashes presented closely in space and time, the binding of two or more modal features may be further enhanced, compared to conditions where subjects attend to the same sounds and flashes alone. If both are asked to attend jointly to the sounds, and jointly ignore the flashes, they may also be less prone to a ventriloquist effect, where the location of the sounds is displaced toward the location of the flashes (Vroomen & De Gelder, 2004).

## Non-visual senses are necessary to extend joint attention

### Jointly attending to invisible sounds or smells

The dominance of vision in the study of, and theorizing about, perception and joint attention may reflect the importance of this modality in humans (Colavita, 1974; Emery, 2000; Itier & Batty, 2009; Sinnett, Spence, & Soto-Faraco, 2007), but should not occult the fact that humans also jointly attend and teach words referring to sounds and smells, not to mention musical features.

Establishing joint attention toward a non-visual target requires access to information about both the other’s attentional focus and, crucially, the target where the other’s attention should be directed. Relative to gaze, a clear limitation of audition and olfaction is that their target of attention is not publicly disclosed to an observer. To establish joint attention coordination on strictly non-visual targets, subjects may be obliged to *indirectly* coordinate on the visual location of these non-visual events and use cognitive strategies to signal and to infer that the target is non-visual. For example, ostensive pointing at the relevant sensory organ (touching one’s ear, or one’s nose) can provide evidence to another agent of the intention of attending to a non-visual stimulus (Baker & Hacker, 2005) (Fig. 2c).

In addition, ostensive strategies could involve *negative* cues such as standing still, and keeping one’s head and eyes motionless to signal that attention should be directed to a non-visual target of joint attention. Here, one prediction would be that such cases would occur only *after* expectations about pointing and gaze have been fully formed – as the strategy rests on using a mismatch between the expectation (that eyes and heads move) and the results (eyes and heads do not move, meaning that the target is non-visible).

Although visual and gestural ostensive cues may be used on some occasions to direct attention to a non-visible target, such behaviors already presuppose that the other agent is capable of understanding that sounds and smells are objects in the world that can be perceived together with others. The developmental onset of the ability to gaze at objects jointly with others is well researched. One outstanding question is when infants start to display an equivalent understanding that others can share with them attention to smells and sounds, and how this understanding is coupled with processing the visual attention of others.

### Jointly attending to amodal features

Gaze-based joint attention enhances basic object recognition, even in very young infants (Cleveland & Striano, 2007; Hoehl, Wahl, Michel, & Striano, 2012; Wahl, Marinović, & Träuble, 2019). However, object-recognition development relies on the ability to perceive global, invariant, and amodal properties like spatial location, tempo, rhythm, and intensity, which can only be conveyed through the combination of different sense modalities (Bahrick & Lickliter, 2014; Hyde et al., 2016). The redundancy introduced by multisensory events can thus be strategically used to establish joint attention on amodal features of objects and events. Bahrick and colleagues suggest that perception of this amodal information is critically important for the development and performance of perceptual object and event recognition (Bahrick, 2010).

One key example of such strategic use is the manner in which the temporal synchrony (when onset, offset, and/or duration of sensory stimuli are the same) between vision and audition can be exploited. For instance, a parent will shake an object in a temporally congruent way with the word they utter, thus enhancing the associating between object and word (Fig. 2d) (Gogate et al., 2000; Gogate & Hollich, 2016; Jesse & Johnson, 2016). The significance of temporal and spatial synchrony across different sensory cues is not only restricted to language learning. Running a toy car over the table or over the infant’s arm while saying “vroom” may not directly lead towards word acquisition, as there is no linguistic element to be acquired. But it may help to bind both visual (e.g., shape) and auditory (e.g., vehicle noises) properties to the same object, the toy car.

The use of two cues highlights an important point. Here the target of joint attention is broader than the cues used to attract and coordinate attention: making a sound while moving a toy-car and looking at it ostensibly can be used to draw attention to the whole multisensory object, including its amodal extension, its weight, texture, etc., and not just its auditory or visual properties.

Conversely, the target of joint attention can be narrower than the object of individual attention and even of mutually shared experiences. For example, while musicians may attend to how others move their bow, hands, and heads, their joint attention is focused on the music they produce or, indeed, an element of the music (a particular voice or a particular theme). Moreover, their auditory joint attention will be coordinated through the gestures of a musical conductor, which provide visual cues about particular aspects in the sounds that musicians must follow – the music’s tempo, for example. In this sense, the target of coordinated attention is narrower than the visual and auditory cues they use to attract and maintain their attention and narrower than the multisensory production that they know they are mutually experiencing.

Taking into account the role of non-visual senses in coordinating attention highlights that the *target of joint attention* can often be different than *the target of each individual’s attention*. Joint attention involves more than merely orienting toward the same target. Perceptual attention can be characterized as the selective information processing of a specific area or features of the sensory world, while ignoring or decreasing processing of other areas and features (Eriksen & James, 1986; Klein & Lawrence, 2012). Joint attention results in a socially mediated enhancement in the processing of sensory information (Mundy, 2018). In other words, joint attention brings about another level of selectivity over an individual’s own perceptual attention. Engaging in joint attention allows us to extract from a fundamentally multisensory experience the relevant integrated targets or specific features (visual, auditory, etc.) for further information processing and social coordination.

### Sensory deficits: Jointly attending to a multisensory object through different senses

What happens when coordination occurs on objects that the two agents experience through different modalities? This is the case when coordinating attention with blind individuals, or individuals whose vision is temporarily blocked (say, they wear opaque glasses). Here, both or at least one agent knows that the other cannot access the object on which attention needs to be coordinated via the visual modality that they themselves use to access the object.

Cases of sensory deprivation (e.g., deafness, blindness, anosmia, hyposmia) provide methodological tools to study the roles of different senses during joint attention, and how individuals with limited sensory access negotiate coordination. Atypical development highlights the manner in which we share attention with others as a function of information access. In a case study of two congenital blind infants, coordinating attention with their caregivers involved auditory information as well as tactile and kinesthetic information, memory, sound changes, air currents, and echolocation (Bigelow, 2003). Deaf-blind children tend to combine two or more sensory sources for coordinating attention toward an object with their non-deaf-blind parents (Núñez, 2014). A 3-year-old child with profound visual and hearing impairment would first draw on touch to check that she has her caregiver’s attention. She would then hold the object of interest towards the caregiver’s face with one hand while continuing to monitor their attention with the other hand, vocalizing excitedly and smiling throughout (Núñez, 2014). Social gaze behavior and joint attention through vision alone can also be impacted by auditory deficits (e.g., Corina & Singleton, 2009; Lieberman, Hatrak, & Mayberry, 2014). There is evidence, for example, that auditory deprivation affects the effect of gaze cues and gaze following. Deaf children (aged between 7 and 14 years old) are more susceptible to the influence of task-irrelevant gaze cues than hearing children (Pavani, Venturini, Baruffaldi, Caselli, & van Zoest, 2019). This effect appears to dissipate in deaf adults, suggesting that the salience of social gaze cues changes during development (Heimler et al., 2015)

These studies reinforce the view that our ability to establish the triadic relation characteristic of joint attention can vary according to the modal pathways used for directing and following the other’s attention (Fig. 2e). In multisensory contexts, agents can share across information to which the other person has no access, or is not actively accessing. To illustrate, suppose we are jointly attending to a coffee cup by vision. In addition, I am also touching the object to judge its temperature. Through our coordinated attention to the cup and by monitoring my responses, you can vicariously gather information on my haptic experience and whether the cup is warm.

## Theoretical implications: Sharing more than a visual world

Philosophers and psychologists have taken the role of joint attention in our understanding of other minds to argue that joint attention is, in fact, essential to understand the concept of a shared objective world, where mind-independent objects are attended in common (Davidson, 1999; Eilan, 2005; Engelland, 2014; Seemann, 2019; Tomasello, 2014). The ability to coordinate attention to an object together with another individual goes hand in hand with the ability to experience the object as a mind-independent entity separate from oneself (Campbell, 2011). This view has pre-eminent precursors in psychology. Lev Vygotsky (2012), in particular, held the doctrine that all higher cognition in an individual arises from an internationalization process of prior social interactions. Vygotsky’s original formulation may seem overly strong, but a Vygotskyan approach has become increasingly influential to account for the social influences observed in the development of cognition and psychiatric disorders (Bolis & Schilbach, 2018; Fernyhough, 2008; Hobson & Hobson, 2011; Tomasello, 2019). Granting that joint attention helps us build a shared objective world, restricting ourselves to gaze and vision alone would make this world incredibly impoverished.

To stress this point, imagine a case where joint attention would *only* occur through gaze-following and looking at pointing gestures: we would only be able to coordinate attention on the visual properties of objects and events. We would certainly be able to learn that most bananas are yellow; we would learn that using color-tinged glasses changes how these properties look; and we would learn that other people may be seeing a drawing upside down when we see it right side up. But how would two people jointly attend to the sound of thunder, or the smell of natural gas? Would they quickly make the difference between pointing at the color of the car, or the car as a whole?

Realizing that we attend to a unitary object or to specific properties cannot occur in a visual-only scenario, or certainly without resorting to more conventional or linguistic means. Using a multisensory combination of cues is necessary to explain that we share an objective world of multisensory objects, sounds, smells, and textures.

## Applications: Multisensory strategies for the clinic, the school, and social robotics

A better understanding of the mechanisms through which multisensory and cross-modal processes help and shape the successful coordination of attention on the same object, or on a given aspect of an object, can have direct implications for several sectors and fields.

### When gaze coordination is limited

In a caregiver-child pair in which one person has a sensory deficit (deaf-blind, deaf, blind), the information that can be shared will be limited in some way, and compensated for in others. Tactile joint attention is crucial for children with visual impairments and multiple sensory disabilities (Chen & Downing, 2006). A child rolling Play-Doh will lead the adult’s hand to share attention to her activity. The adult can follow the child’s lead and focus on what the child is doing by keeping non-controlling tactile contact both with the child’s hands and with the Play-Doh, establishing a reciprocal relation.

An emphasis on gaze interaction, however, can lead to biased assessments of an individual’s ability to coordinate and interact with others. When measured according to vision-based operationalizations, deaf children of hearing parents show a delay in the onset of *visual* joint attentional skills, and symbol-infused joint attention (involving words or symbolic gestures) tends to be less frequent than in typically developing infants (Prezbindowski, Adamson, & Lederberg, 1998). These results have been challenged when factoring the role of other senses: hearing parents do accommodate their deaf children’s hearing status by engaging them via multiple modalities, while parents of typically developing children tend to use alternating unimodal (either visual or auditory) cues during a joint attention episode (Depowski, Abaya, Oghalai, & Bortfeld, 2015). Developmental differences are not pronounced in deaf children of deaf parents, who tend to coordinate attention using both visual and tactile signals (Spencer, 2000).

Taken together, these findings suggest that operationalizations of joint attention based on gaze alone may produce unreliable measures of the real ability of infants to coordinate attention with others. They also show that non-visual senses impinge on the development of joint attention, even for non-visually impaired deaf individuals. Finally, the ability to engage in joint attention depends not just on the atypical infant’s behavior, but, importantly, on that of their caregivers. Adopting a multisensory perspective on joint attention can provide better measures of the development of atypical children and inspire new complementary strategies to foster the development of joint attention skills.

### Multisensory joint attention during learning

The ostensive character of joint attention is central to the acquisition of language (Adamson et al., 2019; Carpenter et al., 1998; Tomasello & Farrar, 1986) and, more generally to the transmission of knowledge and learning (Csibra & Gergely, 2009). In traditional paradigms on the role of joint attention in language development, triadic coordination to a target object is visually established through gaze alternation or pointing, accompanied by the utterance of the linguistic label to be associated with the object (see Akhtar & Gernsbacher, 2007, for a critical overview). As noted above, however, early linguistic development is increasingly recognized as a multisensory process (Gogate & Hollich, 2016; Jesse & Johnson, 2016). Similarly, the importance of multisensory teaching methods is increasingly recognized within pedagogy, both for typically developing children (e.g., Kirkham, Rea, Osborne, White, & Mareschal, 2019; Shams & Seitz, 2008; Volpe & Gori 2019) and for children with learning differences, including dyslexia (e.g., Birsh, 2005) and autistic spectrum disorder (e.g., Mason, Goldstein, & Schwade, 2019).

A better understanding of the interplay of different sense modalities during joint attention, across different ages and neurological conditions, can support the development of multisensory protocols in pedagogical situations. It should also be a reminder of cross-cultural differences when generalizing about teaching: in some cultures, touch, sounds, or smells are more central to social engagement, learning, or communication (Akhtar & Gernsbacher, 2008; Kinard & Watson, 2015). Akhtar and Gernsbacher (2008) review evidence suggesting that in cultures where infants experience continuous physical or vocal contact with their caregivers, and spend less time in face-to-face eye contact, evidence of social engagement will rely on tactile, auditory, and olfactory cues more than mutual gaze cues. Mothers in Kenya, for example, engage in more touching and holding with their infants, and less in eye contact, than mothers in the USA (Richman, Miller, & LeVine, 1992).

### Multisensory joint attention with artificial social agents

The field of social robotics strives to bring artificial agents into hospitals, schools, businesses, and homes – complex social environments that require the enactment of naturalistic non-verbal interactions, including joint attention coordination (Clabaugh & Matarić, 2018; Kaplan & Hafner, 2006; Yang et al., 2018). For a robot to help a human partner assemble a piece of furniture, stack blocks with children in the playground, and assist people with disabilities in their daily lives, they need to be sensitive to what the human is attending to, and asking them to attend to.

Whether an artificial agent can successfully engage in joint attention with humans will depend on how well they can meet the behavioral expectations of their human interaction partner. Will they be able to both initiate and follow attentional cues in a naturalistic manner (Pfeiffer-Leßmann, Pfeiffer, & Wachsmuth, 2012)? One current approach is to enable social robots to mimic human gaze behaviors (Admoni & Scassellati, 2017; Kompatsiari, Ciardo, Tikhanoff, Metta, & Wykowska, 2019). However, while human participants do respond to the gaze of artificial agents (Willemse, Marchesi, & Wykowska, 2018), they are also highly sensitive to momentary multimodal behaviors produced by their artificial partner (Yu, Schermerhorn, & Scheutz, 2012). By adopting a multisensory perspective on human-robot joint attention, it is possible to examine non-visual cues emitted by the artificial agent, so that they accord with the expectations of human interaction partners. Being sensitive to the non-visual cues emitted by humans could also improve the spatial and temporal resolution of attention-orienting in robots.

## Conclusion

Any episode of visual attention will, de facto, rely on background multisensory processing: we rely on proprioceptive and vestibular cues to visually orient our attention and ourselves in the world. Multisensory interactions, however, play a more substantial role in the coordination of attention across social agents: infants and adults recruit multiple sense modalities to initiate and follow someone’s attention to a specific object or location in space. These interactions can be distinguished depending on whether they facilitate the coordination of visual attention, or whether they extend the coordination to non-visual and amodal properties. While non-visual modalities are useful complements for vision in the former case, they are essential in the latter case: some kinds of joint attention are necessarily multisensory, and could not be carried by vision alone.

This multisensory approach has implications for behavioral and developmental models of joint attention. Just as selective attention can be described as a cognitive capacity that both influences and is influenced by perceptual processes across different modalities, models of joint attention must be flexible enough to incorporate how it relies on dynamic information from multiple senses. It also has practical implications to overcome clinical deficits in joint attention, augment its pedagogical role, and address the challenge of coordinating attention between humans and social robots.

### Author Note

MF was supported in part by funds from LMU Munich‘s Institutional Strategy LMUexcellent within the framework of the German Excellence Initiative. OD is supported by the NOMIS foundation “Dise” grant.
